# Injectable and Temperature-Sensitive Titanium Carbide-Loaded Hydrogel System for Photothermal Therapy of Breast Cancer

**DOI:** 10.3389/fbioe.2021.791891

**Published:** 2021-12-23

**Authors:** Jun Yao, Chuanda Zhu, Tianjiao Peng, Qiang Ma, Shegan Gao

**Affiliations:** ^1^ Henan Key Laboratory of Cancer Epigenetics, Cancer Institute, The First Affiliated Hospital, College of Clinical Medicine of Henan University of Science and Technology, Luoyang, China; ^2^ School of Basic Medical Sciences, Peking University Health Science Center, Beijing, China; ^3^ Institute of Environment and Sustainable Development in Agriculture, Chinese Academy of Agricultural Sciences, Beijing, China

**Keywords:** pluronic F127 hydrogel, photothermal therapy, anti-cancer, thermosensitive, Ti_3_C_2_ nanoparticles

## Abstract

Recently, organic–inorganic hybrid materials have gained much attention as effective photothermal agents for cancer treatment. In this study, Pluronic F127 hydrogel-coated titanium carbide (Ti_3_C_2_) nanoparticles were utilized as an injectable photothermal agent. The advantages of these nanoparticles are their green synthesis and excellent photothermal efficiency. In this system, lasers were mainly used to irradiate Ti_3_C_2_ nanoparticles to produce a constant high temperature, which damaged cancer cells. The nanoparticles were found to be stable during storage at low temperatures for at least 2 weeks. The Ti_3_C_2_ nanoparticles exhibited a shuttle-shaped structure, and the hydrogels presented a loosely meshed structure. In addition, Ti_3_C_2_ nanoparticles did not affect the reversible temperature sensitivity of the gel, and the hydrogel did not affect the photothermal properties of Ti_3_C_2_ nanoparticles. The *in vitro* and *in vivo* results show that this hydrogel system can effectively inhibit tumor growth upon exposure to near-infrared irradiation with excellent biocompatibility and biosafety. The photothermal agent-embedded hydrogel is a promising photothermal therapeutic strategy for cancer treatment by enhancing the retention *in vivo* and elevating the local temperature in tumors.

## Introduction

Photothermal therapy (PTT) has been widely used in cancer therapy because of its excellent antitumor effects (Senapati et al., 2018; Zhi et al., 2020). In PTT, a near-infrared (NIR: 700–1,100 nm) laser is used to irradiate the target area where a PTT agent is present. This produces a constant high temperature (40°C–50°C), which either induces the death of local cancer cells or increases their sensitivity to other therapies (Chen et al., 2017; Yu et al., 2017; Zhao et al., 2018; Lu et al., 2020). PTT has shown significant therapeutic effects in various antitumor studies (Jiang et al., 2018; Zhu et al., 2018; Chen et al., 2019a). Many photothermal agents (PTAs) have been developed to convert the energy of NIR lasers into heat for cancer research, including organic dye molecules (Sahu et al., 2016), precious metal materials (Xu et al., 2019), carbon-based materials (Liu et al., 2019a; Rafieerad et al., 2019), and other inorganic materials (Popescu et al., 2011). PTA is often administered intravenously (Popescu et al., 2011; Jiang et al., 2018; Zhang et al., 2018; Zhi et al., 2020). However, there are some concerns regarding the toxicity of intravenously administered PTAs. Most PTAs contain heavy metals. In addition, the inherent instability of organic agents limits their therapeutic effects. For example, indocyanine green often undergoes light bleaching and is rapidly eliminated after intravenous administration (Sahu et al., 2016; Chen et al., 2017; Yu et al., 2017).

Therefore, nanomaterials with good photothermal stability have been designed to overcome the limitations of traditional PTAs by using an NIR laser to increase the temperature (Chen et al., 2019b; He et al., 2019; Xu et al., 2019; Zhao et al., 2019; He et al., 2020; Wang et al., 2020). As an outstanding nanomaterial, titanium carbide (Ti_3_C_2_) shows good performance in the anticancer field of PTT. For example, Ti_3_C_2_ can absorb large amounts of light and shows high light-thermal conversion efficiency and high biocompatibility at NIR wavelengths. Photothermal nanomaterials can be actively concentrated in tumor tissues by modifying the nanoparticle surface to avoid systemic toxicity (He et al., 2019; Zhao et al., 2019). The treatment conditions (laser power, exposure positions, and exposure times) can be artificially controlled to minimize damage to the surrounding healthy tissue. In addition, photothermal nanomaterials can be actively targeted to tumor sites using ultrasonic or magnetic guidance (Senapati et al., 2018; Chen et al., 2019b). However, nanomaterials with targeted functions not only require costly materials and complex operations but also have a short half-life in the body (Xu et al., 2019; He et al., 2020; Liu et al., 2020; Lu et al., 2020). Thus, a simple and effective local slow-release administration system for PTT is urgently needed.

Currently, commonly used intratumoral or peritumoral sustained-release delivery systems include microneedles (Yu et al., 2020) and hydrogels (Fu et al., 2019; Geng et al., 2020), which can prolong drug release into the targeted tissue. For example, *in situ* gels contain a drug loaded in a specific polymer carrier. *In situ* gels adopt a sol form *in vitro* and quickly form a gel after administration *in vivo*, prolonging drug retention in the tumor (Liu et al., 2017; Geng et al., 2020). The gel formation mechanism can be illustrated by Pluronic F127 (F127), a three-segment copolymer that consists of polyoxymethylene and polyoxypropylene. At low temperatures, F127 exists as a single molecule. As the temperature increases, the hydrophobic fragments of polyoxypropylene in the F127 molecule dehydrate to form spherical gel beams consisting of an inner core of hydrophobic polyoxypropylene and external swollen polyoxyethylene. Subsequently, the stacked bundles further wind to form a gel (Chen et al., 2019a; Russo and Villa, 2019; Yu et al., 2021). As a mild-sensitized pharmaceutical accessory, F127 limits the random dispersion of nanoparticles within tissues to avoid damage to normal tissues (Fu et al., 2019).

To effectively treat breast cancer and reduce the side effects of traditional PTA, we designed an injectable and temperature-sensitive F127 hydrogel mixed with Ti_3_C_2_ nanoparticles. The Ti_3_C_2_ nanoparticles and temperature-sensitive F127 hydrogel jointly construct the Ti_3_C_2_-Gel system, which is administered by local injection at low temperatures and forms a gel at 37°C in the body. The results of *in vivo* and *in vitro* experiments showed that the system had an excellent antitumor effect. In general, the combination of Ti_3_C_2_ nanoparticles and temperature-sensitive hydrogels is a promising antitumor local administration system with the potential for use in clinical cancer treatments.

## Materials and Methods

### Materials

Ti_3_C_2_ MXene solution was purchased from Beike 2D Materials Co., Ltd. (Beijing, China). Pluronic F127 was purchased from Sigma (St. Louis, MO, USA). Dulbecco’s modified Eagle’s medium (DMEM) and fetal bovine serum were purchased from Gibco (Grand Island, NY, USA). The Cell Counting Kit-8 (CCK-8) was obtained from Dojindo Laboratories (Kumamoto, Japan). The hematoxylin–eosin staining kit (H&E) and terminal deoxynucleotidyl transferase-mediated dUTP nick end labeling (TUNEL) kit were purchased from Solarbio (Beijing, China). All other reagents were analytically pure and used without further purification. Deionized water was used in this study.

### Cell and Mice

Mouse breast cancer cells (4T1 cells) were obtained from the American Type Culture Collection (Manassas, VA, USA) and cultured in DMEM containing 1% penicillin–streptomycin and 10% fetal bovine serum at 37°C under a 5% CO_2_ atmosphere. Female BALB/c mice (6–8 weeks of age) were obtained from Charles River Laboratories (Wilmington, MA, USA) for animal experiments. All animal experiments were approved by the Animal Care and Use Committees at the First Affiliated Hospital and College of Clinical Medicine of Henan University of Science and Technology.

### Preparation and Characterization of Ti_3_C_2_-Gel

**FIGURE 1:**
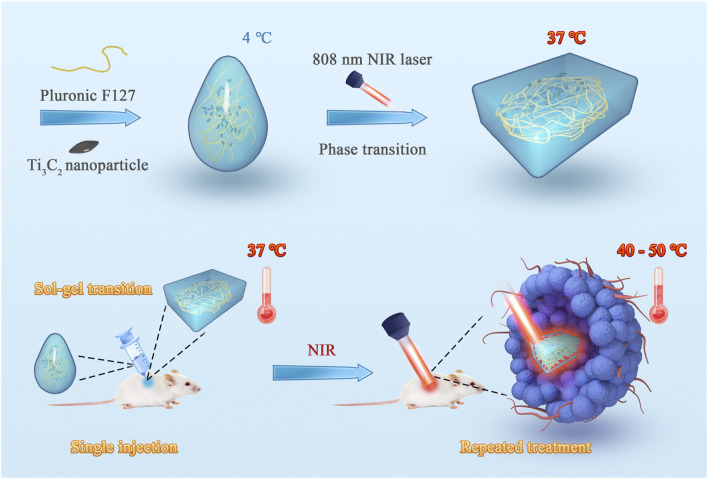
Schematic illustration of the fabrication of the injectable hydrogel system with excellent photo–heat transition capacity. This system was fabricated using a one-step synthesis method. Ti_3_C_2_ nanoparticles and thermosensitive Pluronic F127 were mixed to prepare the photothermal agent-embedded hydrogel system. The system can form *in situ* gel in tumor tissue through sol–gel transition and prolong the retention of Ti_3_C_2_ nanoparticles. Using near-infrared laser irradiation, repeated treatments can be achieved with a single injection.

The Ti_3_C_2_-Gel system was fabricated using a simple mixture. Ti_3_C_2_ nanoparticles were obtained through ultrasonic treatment of Ti_3_C_2_ MXene solution. The Ti_3_C_2_ nanoparticles and thermosensitive Pluronic F127 were mixed to prepare the PTA-embedded hydrogel system (Figure 1). The F127 powder was dissolved in water to form hydrogels with different concentrations (40%, 35%, 30%, 25%, 20%, 19%, and 18%). To obtain different final concentrations of Ti_3_C_2_ nanoparticles embedded in F127 hydrogels (Ti_3_C_2_: 400, 200, 100, 50, and 25 μg/ml; F127 hydrogels: 20%), a solution of Ti_3_C_2_ nanoparticles was added to the 20% F127 hydrogels. These hydrogels (including Gel and Ti_3_C_2_-Gel) were stored at 4°C until use. After dilution by 50-fold with pure water, the size and zeta potential of these samples (Ti_3_C_2_ and Ti_3_C_2_-Gel) were measured by dynamic light scattering (PSS ZPW 388, Nicomp, Orlando, FL, USA). The appearance of these samples was recorded using transmission electron microscopy (JEOL174 1200EX, Tokyo, Japan).

To determine the phase change temperature of the hydrogels, different concentrations of blank F127 gel (40%, 35%, 30%, 25%, 20%, 19%, 18%) and Ti_3_C_2_-Gel (Ti_3_C_2_: 50 μg/ml) were added to glass bottles and placed at 4°C to maintain the liquid state to remove bubbles from the samples. During this assay, an agitator was placed in the bottles, which were placed in a water bath under a tunable temperature and magnetic stirring. The heating rate was 0.5°C/min, and the rotation rate of the magnetic agitator was 300 rpm. The temperature was recorded as the gel formation temperature when the agitator was completely stopped. Each sample was measured three times.

In addition, the rheumatic behaviors of blank F127-Gel (20%) and Ti_3_C_2_-Gel (Ti_3_C_2_: 50 μg/ml; 20% F127 Gel) were observed using a rheometer (MCR92, Anton Paar, Graz, Austria). These samples were placed on the plate of the rheometer, and silicone oil was added to the outer edge of the samples to prevent moisture evaporation.

To study the stability of the Ti_3_C_2_ nanoparticles and hydrogel sample, Ti_3_C_2_ nanoparticles (50 μg/ml) and Ti_3_C_2_-Gel (Ti_3_C_2_: 50 μg/ml; 20% F127 Gel) were stored at 4°C. The samples were diluted with pure water before and after storage. Changes in the size of the Ti_3_C_2_ nanoparticles and Ti_3_C_2_-Gel were measured using a particle sizing system.

### Assessment of Photothermal Properties

To evaluate the photothermal conversion capability of Ti_3_C_2_ nanoparticles aqueous solution and Ti_3_C_2_-Gel, 1-ml samples were separately added to different centrifuge tubes and then exposed to an 808-nm NIR laser (1 W/cm^2^). A thermal imaging system (FOTRIC, Allen, TX, USA) was used to record the changes in temperature of the samples.

To further evaluate the stability of photothermal conversion, 1 ml Ti_3_C_2_ nanoparticle solution (Ti_3_C_2_: 50 μg/ml) and Ti_3_C_2_-Gel (Ti_3_C_2_: 50 μg/ml; 20% F127 Gel) were separately placed into different tubes and then exposed to the NIR laser (1 W/cm^2^). After the samples had naturally cooled to room temperature (20°C–30°C), they were repeatedly exposed to laser light. We used thermal imaging cameras to record the temperature increase in the samples, which was used to plot a curve of photothermal stability.

### *In vitro* Cytotoxicity

The cytotoxicity of Ti_3_C_2_ was evaluated using the standard CCK-8 assay. 4T1 cells were seeded at a density of 5 × 10^3^ cells/well in 96-well plates (37°C, 5% CO_2_). After 12 h of incubation, various concentrations of Ti_3_C_2_ (400, 200, 100, 50, and 25 μg/ml) were dispersed into fresh DMEM and inoculated into the wells. Cells without Ti_3_C_2_ were used as controls. After incubation for another 24 h, the cells were washed with PBS three times. The absorbance of the cells was measured at 450 nm using a microplate reader. Each group was analyzed in triplicate.

To evaluate the photothermal therapeutic effects of Ti_3_C_2_ *in vitro* on tumor cells, 4T1 cells were seeded into 96-well plates (5 × 10^3^ cells/well) and incubated overnight at 37°C in a 5% CO_2_ atmosphere. Next, Ti_3_C_2_ nanoparticles were added to the wells at final concentrations of 25, 50, 100, 200, and 400 μg/ml. The cells were exposed to an 808-nm NIR laser (1 W/cm^2^) for 1 min, with untreated 4T1 cells used as a control. The cells were incubated for a further 24 h. The Ti_3_C_2_ nanoparticles were removed and washed with PBS. Cell viability was measured at 450 nm following the instructions for CCK-8.

### *In vivo* Antitumor Performance

To prepare a 4T1 tumor-bearing mouse model, 1 × 10^6^ 4T1 cells were administered subcutaneously into the right armpit of BALB/c mice (female, 6–8 weeks old). When the tumor volume reached approximately 100 mm^3^, 4T1 tumor-bearing mice were used for *in vivo* therapy experiments. The mice were randomly divided into nine groups (*n* = 5/group): mock (untreated), PBS, Pluronic F127 Gel, Ti_3_C_2_, Ti_3_C_2_-Gel, PBS + NIR, Gel + NIR, Ti_3_C_2_ +NIR, and Ti_3_C_2_-Gel + NIR (Ti_3_C_2_: 50 μg/ml; Pluronic F127 Gel: 20%). At day 1, 30 µl of different preparations was administered into the tumor in mice. After the mice were anesthetized, the tumors were exposed to an 808-nm NIR laser at 1 W/cm^2^ for 2 min on days 1, 3, and 5. The length and width of the tumors were measured every other day. Moreover, the body weights of mice were recorded to map the curve of weight–time changes. The tumor volume was calculated according to the following formula: volume = length × (width^2^)/2. All mice were sacrificed on day 15, and the tumors were collected to evaluate the therapeutic effect of PTT *in vivo.*

### *In vivo* Safety Evaluation

Blood was collected from anesthetized mice, and the blood samples were placed at room temperature for 2 h and centrifuged at 1,000 × g for 20 min to isolate the serum. Blood biochemistry indicators were detected using a BS-180 automatic biochemical analyzer (Mindray, Shenzhen, China). The tumors were collected for H&E and TUNEL staining to evaluate the toxicity of the treatment.

### Statistical Analysis

Quantitative data are expressed as the mean ± standard deviation. Statistical analyses were performed using GraphPad Prism version 8.0 software (GraphPad, Inc, La Jolla, CA, USA). Student’s *t*-test and one-way analysis of variance were used to test the significance of differences. Statistical significance was set at *p* < 0.05 (*p < 0.05, **p < 0.01).

## Results and Discussion

### Characterization of Ti_3_C_2_ and Ti_3_C_2_-Gel

**FIGURE 2:**
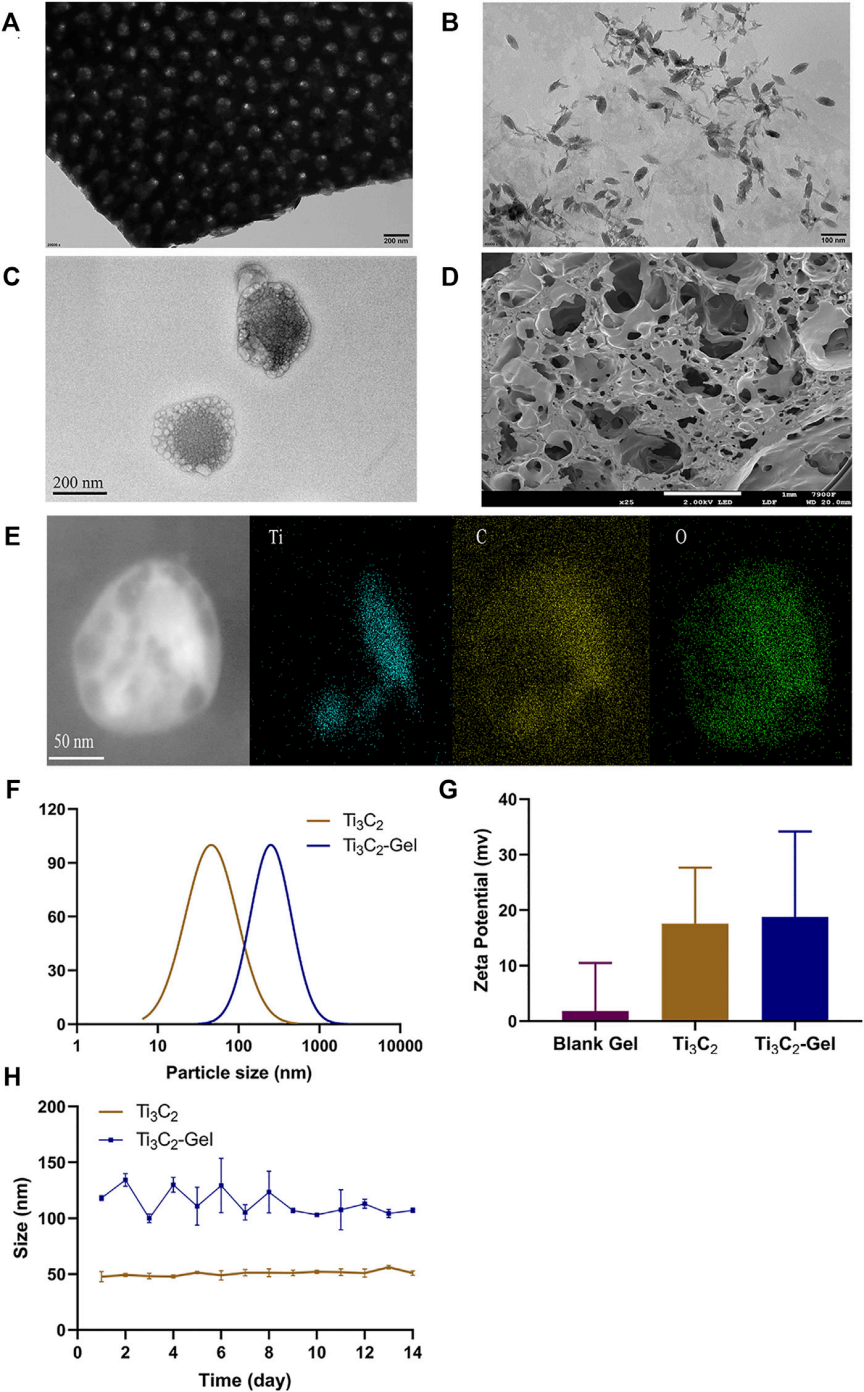
Preparation and characterization of Ti_3_C_2_ nanoparticles and Ti_3_C_2_-Gel. Transmission electron microscopy image of **(A)** Ti_3_C_2_ MXene solution (scale bar: 200 nm), **(B)** Ti_3_C_2_ nanoparticles (scale bar: 100 nm), and **(C)** Ti_3_C_2_-Gel (scale bar: 200 nm). **(D)** Scanning electron microscopy image of Pluronic F-127 gel (scale bar: 1 mm). **(E)** Energy-dispersive X-ray image of Ti_3_C_2_-Gel (scale bar: 50 nm). **(F)** Particle sizes of Ti_3_C_2_ nanoparticles and Ti_3_C_2_-Gel. **(G)** Zeta potentials of the different preparations. **(H)** Stability of Ti_3_C_2_ and Ti_3_C_2_-Gel sizes.

As shown in Figure 2A, the Ti_3_C_2_ MXene solution existed as a two-dimensional flaky substance. After ultrasonic destruction, the flaky structure disintegrated into uniform shuttle-shaped nanoparticles with a size of nearly 50–100 nm, as revealed by transmission electron microscopy (Figure 2B). The particle size of the Ti_3_C_2_-Gel was approximately 100–200 nm, which was larger than that of the Ti_3_C_2_ nanoparticles (Figure 2C). The results in Figure 2C show that the F127 hydrogels were coated on the surface of the Ti_3_C_2_ nanoparticles. Scanning electron microscopy images showed that the blank F127 hydrogels had a loose and porous structure along with a three-dimensional network (Figure 2D). The main elemental compositions of the Ti_3_C_2_-Gel were Ti, C, and O, suggesting the formation of composites (Figure 2E). Therefore, F127 hydrogels have the potential to easily accommodate large amounts of nanoparticles. In addition, the particle sizes of Ti_3_C_2_ and Ti_3_C_2_-Gel were measured using dynamic light scattering, which corroborated the scanning electron microscopy results (Figure 2F). All zeta potential values of the Ti3C_2_ nanoparticles, F127 hydrogels, and Ti_3_C_2_-Gel were positive. To assess the stability of the PTA, the Ti_3_C_2_ nanoparticles and Ti_3_C_2_-Gel were stored at 4°C for 2 weeks and examined for changes in particle size (Figure 2H). The results showed that hydrogels are excellent drug reservoirs, and the samples had a stable particle size, indicating that they are suitable for long-term storage.

### Temperature-Sensitive Characteristics of Ti_3_C_2_-Gel

**FIGURE 3:**
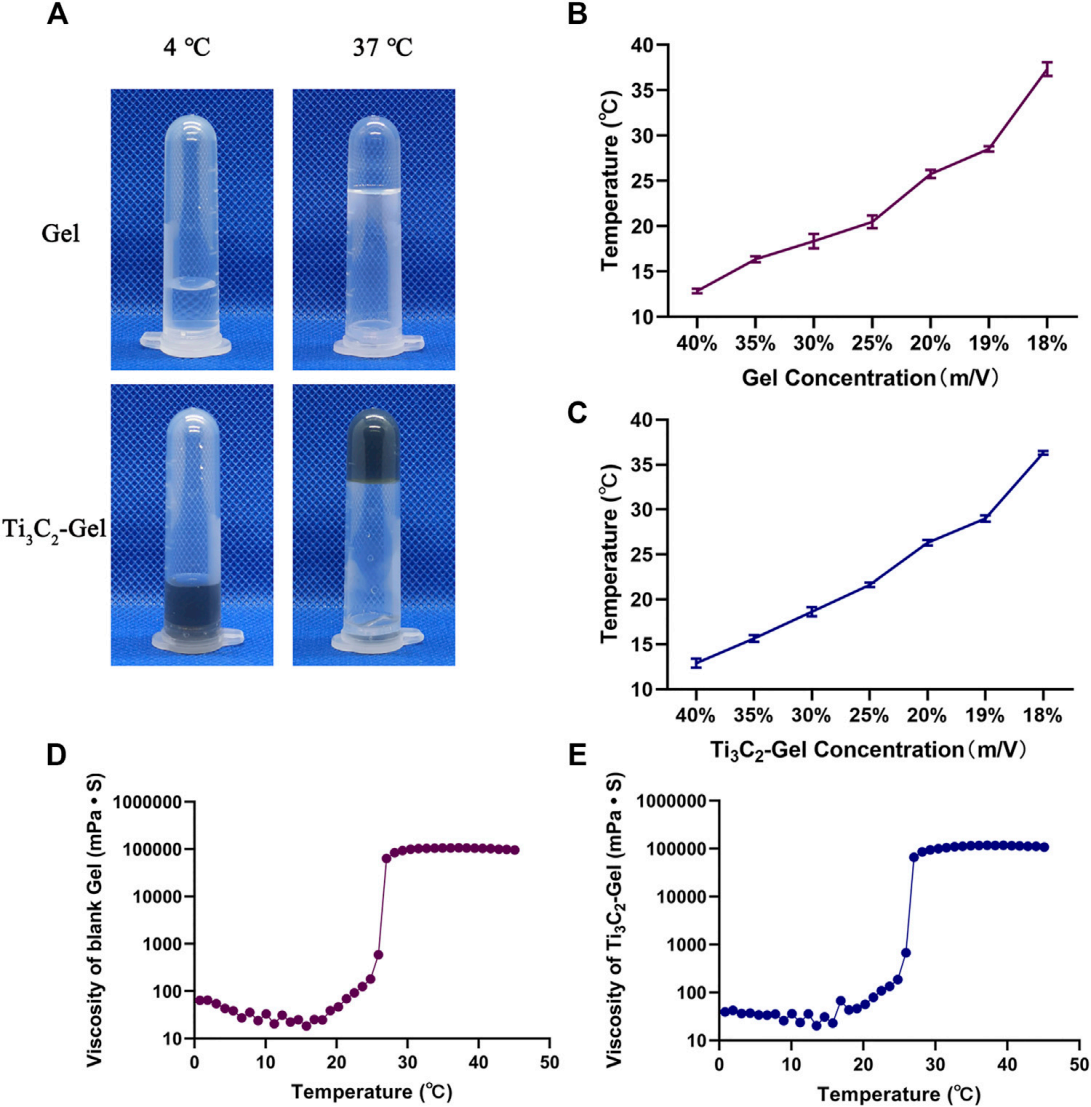
Assessment of photothermal properties. **(A)** Morphology of Pluronic F127 gel and Ti_3_C_2_-gel at 4°C and 37°C. **(B,C)** Phase transition temperature of Pluronic F127 gel and Ti_3_C_2_-Gel (concentration of Ti_3_C_2_ was 50 μg/ml). **(D,E)** Rheological properties of Pluronic F127 and Ti_3_C_2_-gel (concentration of Pluronic F127 was 20%, and that of Ti_3_C_2_ was 50 μg/ml).

Figure 3A shows images of the blank F127 hydrogel and Ti_3_C_2_-Gel at two different temperatures (4°C and 37°C). Both samples were liquids at low temperatures and presented injectable features. They changed to semisolids at 37°C and did not flow even when inverted, which may prolong the retention time of the Ti_3_C_2_ nanoparticles in the tissue. By test tube inversion in a water bath at 37°C, the time required for the gels to convert from a sol to a gel was 21 ± 1 s. Doping nanoparticles in the gel did not affect the reversible sol–gel transition of the F127 hydrogel. Observation of the samples showed that the nanoparticles were evenly distributed in the F127 hydrogel matrix. To further explain the temperature-sensitive characteristics of the F127 hydrogels, *in vitro* experiments were conducted to determine the phase transition temperature and rheological performance of the hydrogels. The phase transition temperature is the temperature at which the hydrogel changes from a sol state to a gel state and is among the most important evaluation indicators for temperature-sensitive hydrogels. The phase transition temperatures of the F127 hydrogels differed at different concentrations. As shown in Figures 3B,C, the phase transition temperatures of F127 hydrogels and Ti_3_C_2_-Gel increased with decreasing concentrations, and Ti_3_C_2_ had no obvious effect on the phase transition temperature of F127 hydrogels. When the concentration of F127 hydrogels was 18% and 19%, the phase transition temperatures were 36.3 ± 0.245°C and 29.0 ± 0.294°C, respectively. Although the phase transition temperature at an F127 hydrogel concentration of 18% was closer to the human body temperature, in the experiment, 18% and 19% F127 hydrogels were observed to form a second sol state at temperatures greater than 40°C. When the concentration of F127 hydrogels was less than 20%, the kinetic energy of the colloidal particles increased with increasing temperature and accelerated movement speed. Thus, the gel state was unstable, and a concentration of 20% F127 hydrogel was the best choice. It is beneficial for the blank hydrogel and Ti_3_C_2_-Gel to pass through the syringe needle in a sol state during injection and then quickly transform into a gel state in the body.

Next, the complex rheological performances of the blank hydrogel and Ti_3_C_2_-Gel were detected using a rheometer to draw temperature–viscosity curves (Figures 3D,E). The viscosity profiles of the blank hydrogel and Ti_3_C_2_-Gel showed similar viscosity characteristics, with a phase transition temperature of 25.4°C, indicating that the Ti_3_C_2_ nanoparticles had negligible effects on the temperature-sensitive performance of F127 hydrogels. The viscosity of the gels was positively correlated with increased temperature. In the phase transition temperature range, the viscosities of the two formulations increased significantly. The F127 hydrogel was in a sol state, with a viscosity of less than 100 mPa s, ensuring that the entire system was injectable. When the temperature rose to 27°C, the F127 hydrogel adopted a gel state with a stable viscosity higher than 10,000 mPa s, indicating that a drug reservoir could be formed in the body after local administration.

### *In vitro* Photothermal Performance

**FIGURE 4:**
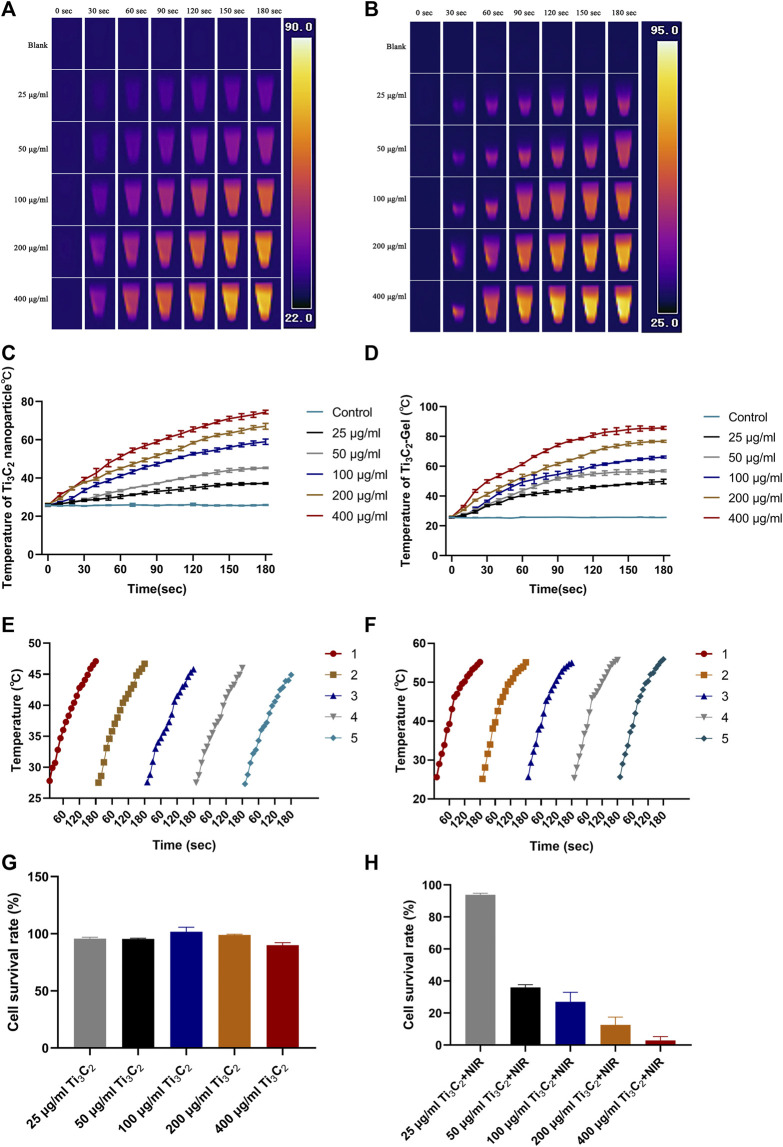
*In vitro* photothermal performance. **(A,B)** Temperature diagram of Ti_3_C_2_ nanoparticles and Ti_3_C_2_-Gel under 808-nm irradiation (1 W/cm^2^). **(C,D)** Heating curve of different concentrations of Ti_3_C_2_ nanoparticles and Ti_3_C_2_-Gel under 808-nm irradiation (1 W/cm^2^). **(E,F)** Heating curves of Ti_3_C_2_ nanoparticles and Ti_3_C_2_-Gel treated with repeated 808-nm irradiation (1 W/cm^2^). **(G)** Viabilities of 4T1 cells incubated with different concentrations of Ti_3_C_2_ nanoparticles in DMEM media for 12 h. **(H)** Cytotoxicity of Ti_3_C_2_ nanoparticles against 4T1 cells exposed to the 808-nm NIR laser (1 W/cm^2^) for 1 min (*n* = 4/group).

To evaluate the photothermal performance of this hydrogel system, Ti_3_C_2_ nanoparticles and Ti_3_C_2_-Gel were exposed to an 808-nm laser under different conditions (Figures 4A–D). It has been reported that a power of 1 W/cm^2^ is the most commonly used safe and effective range (Chen et al., 2019b; Fu et al., 2019; Zhi et al., 2020). The images and temperature changes of these samples were recorded using a thermal imaging system. Based on the heating curve and images, Ti_3_C_2_ and the hydrogel system showed excellent photothermal properties. Under the same irradiation power, the increase in the temperature of samples in the tube was concentration- and time-dependent. In addition, the temperature of the Ti_3_C_2_ nanoparticle solution (50 μg/ml) reached 40°C, whereas that of the Ti_3_C_2_-Gel system reached 50°C after exposure to the NIR laser for 2 min. The Ti_3_C_2_ nanoparticles without encapsulation in the hydrogel were so dispersed that the heat generated was more likely to be lost, whereas the gel system was conducive to the accumulation of Ti_3_C_2_ nanoparticles. In addition, the blank hydrogel exhibited negligible temperature changes under NIR laser exposure.

An excellent PTA must produce constant high temperatures to facilitate repeated photothermal treatment (Figures 4E,F). To evaluate photothermal stability, the Ti_3_C_2_ nanoparticle solution (50 μg/ml) and Ti_3_C_2_-Gel (Ti_3_C_2_: 50 μg/ml; Pluronic F127 Gel: 20%) were tested by recording heating curves with irradiation (808 nm, 1 W/cm^2^). The temperature of both Ti_3_C_2_ nanoparticles and Ti_3_C_2_-Gel rapidly reached 45°C upon NIR irradiation. The photothermal performance of the Ti_3_C_2_ nanoparticles and Ti_3_C_2_-Gel remained constant over five cycles of repeated laser irradiation, indicating the potential of Ti_3_C_2_-Gel to act as a durable PTA for cancer treatment. The Ti_3_C_2_-Gel system may achieve multiple treatment effects following a single injection.

Based on the above results, the toxicity and photothermal therapeutic efficacy of Ti_3_C_2_ on 4T1 cells were investigated in a CCK-8 assay. Figures 4G,H show the survival rate of 4T1 cells against different treatments for 24 h. As observed in the CCK-8 assay, Ti_3_C_2_ nanoparticles at different concentrations (25, 50, 100, 200, and 400 μg/ml) had no obvious lethal effects on 4T1 cells. The cell viability rates were all above 90%, suggesting negligible toxicity of the Ti_3_C_2_ nanoparticles. However, cell death was observed after laser exposure (808 nm, 1 W/cm^2^, 1 min). The survival rate of cells varied with the increase in the concentration of Ti_3_C_2_ nanoparticles under the same laser output power. As the concentration of Ti_3_C_2_ nanoparticles increased, the survival rate of the cells decreased. At a concentration of 50 μg/ml, most tumor cells were killed, exhibiting an excellent therapeutic effect. Generally, a high concentration of exogenous nanomaterials increases metabolic pressure on animals. To prepare materials economically and safely, and from the perspective of animal welfare, we chose the lowest effective drug concentration.

### *In vivo* Antitumor Performance

**FIGURE 5:**
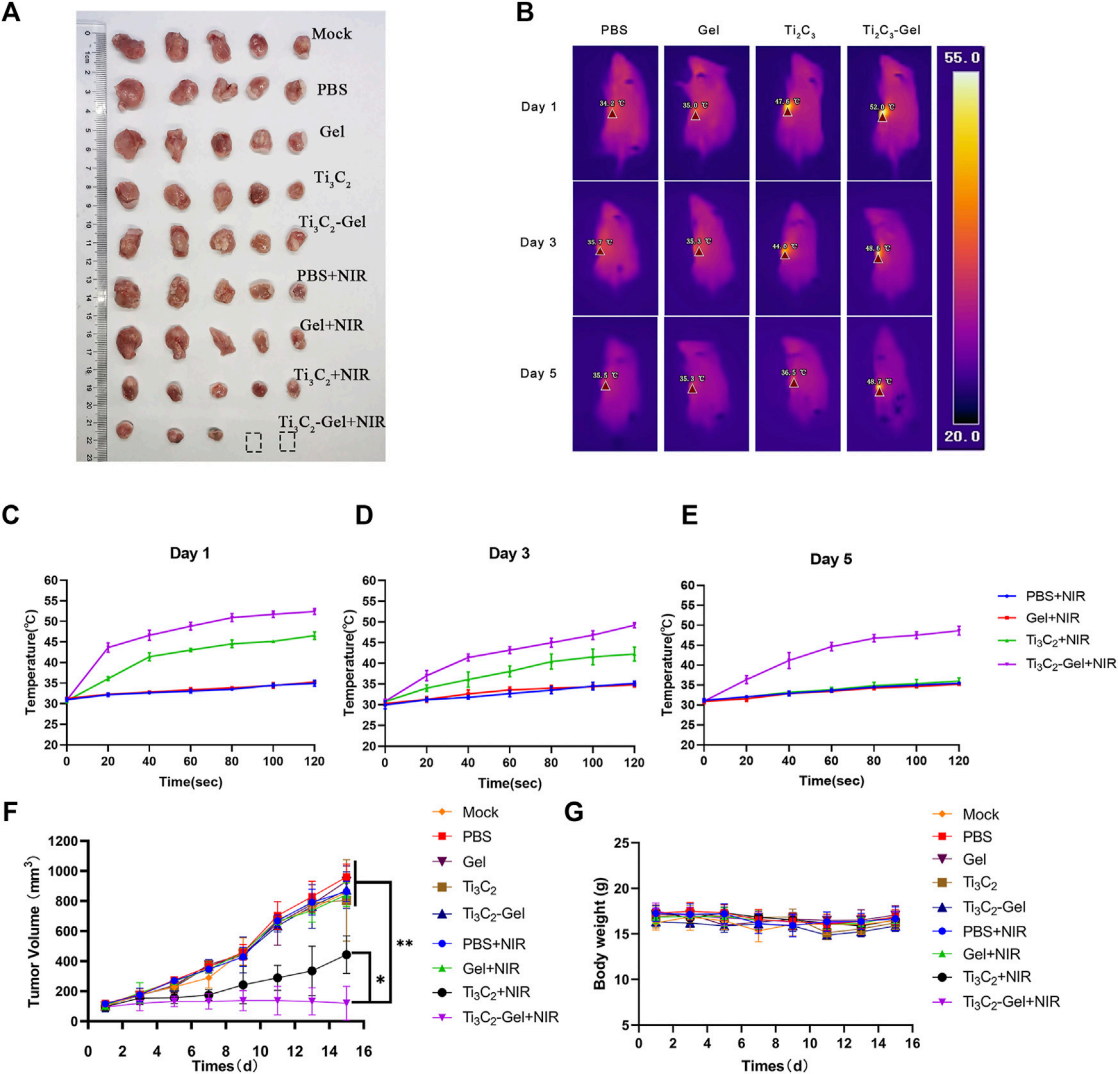
*In vivo* antitumor performance. **(A)** Digital images of tumors of mice after treatment. **(B)** Thermal images of mice irradiated with an 808-nm laser. **(C–E)** Temperature in tumor-bearing mice recorded by a thermal imager under the 808-nm laser. **(F)** Growth curves of tumors in all mice after treatment. *n* = 5/group, mean ± SD, **p < 0.01. **(G)** Average body weight of mice in different groups.

Encouraged by the above results, we further evaluated the photothermal antitumor efficacy of Ti_3_C_2_-Gel in 4T1 tumor-bearing mouse models. After the tumors reached 100 mm^3^ in size, the 4T1 tumor-bearing mice were randomly divided into nine groups: mock, PBS, Pluronic F127 Gel, Ti_3_C_2_ aqueous solution, Ti_3_C_2_-Gel, PBS + NIR, Pluronic F127 Gel + NIR, Ti_3_C_2_ aqueous solution + NIR, and Ti_3_C_2_-Gel + NIR. To determine the advantages of the gel system, we used thermal imaging to record the temperature generated upon laser exposure. NIR treatment was performed on days 1, 3, and 5; at 2 weeks after treatment, all tumor tissues were collected to evaluate antitumor efficacy. Notably, upon treatment with Ti_3_C_2_+NIR and Ti_3_C_2_-Gel + NIR, tumor growth was significantly suppressed (Figures 5A,F). The tumor tissues treated with the Ti_3_C_2_ nanoparticles and Ti_3_C_2_-Gel were smaller than those of the control groups. Using a thermal camera, we observed that the Ti_3_C_2_+NIR group reached the desired therapeutic temperature upon irradiation at 808 nm at the beginning of the experiment. However, the nanoparticles did not reach the initial high temperature over time, similar to the observations in the other control groups. In contrast, Ti_3_C_2_+NIR treatment led to a high temperature during the long treatment period (Figures 5B–E). Interestingly, some tumor tissues faded away at the end of treatment with Ti_3_C_2_-Gel and NIR radiation. During the treatment, the temperature of the tumor tissues increased to 50°C within 2 min, which can disrupt the cell membranes of cancer cells; the underlying mechanisms resulting from PTT have been reported as follows: 1) inhibition of DNA, RNA, and protein synthesis at high temperatures; 2) changes in cell membrane permeability; 3) induction of immunogenic death of cancer cells due to the release of tumor-specific antigens; and 4) production of blood vessel spasms that result in intravascular thrombosis of tumor tissue (Liu et al., 2019b; Poursalehi et al., 2019; Ye et al., 2019; Shang et al., 2020; Chang et al., 2021; Li et al., 2021).

**FIGURE 6:**
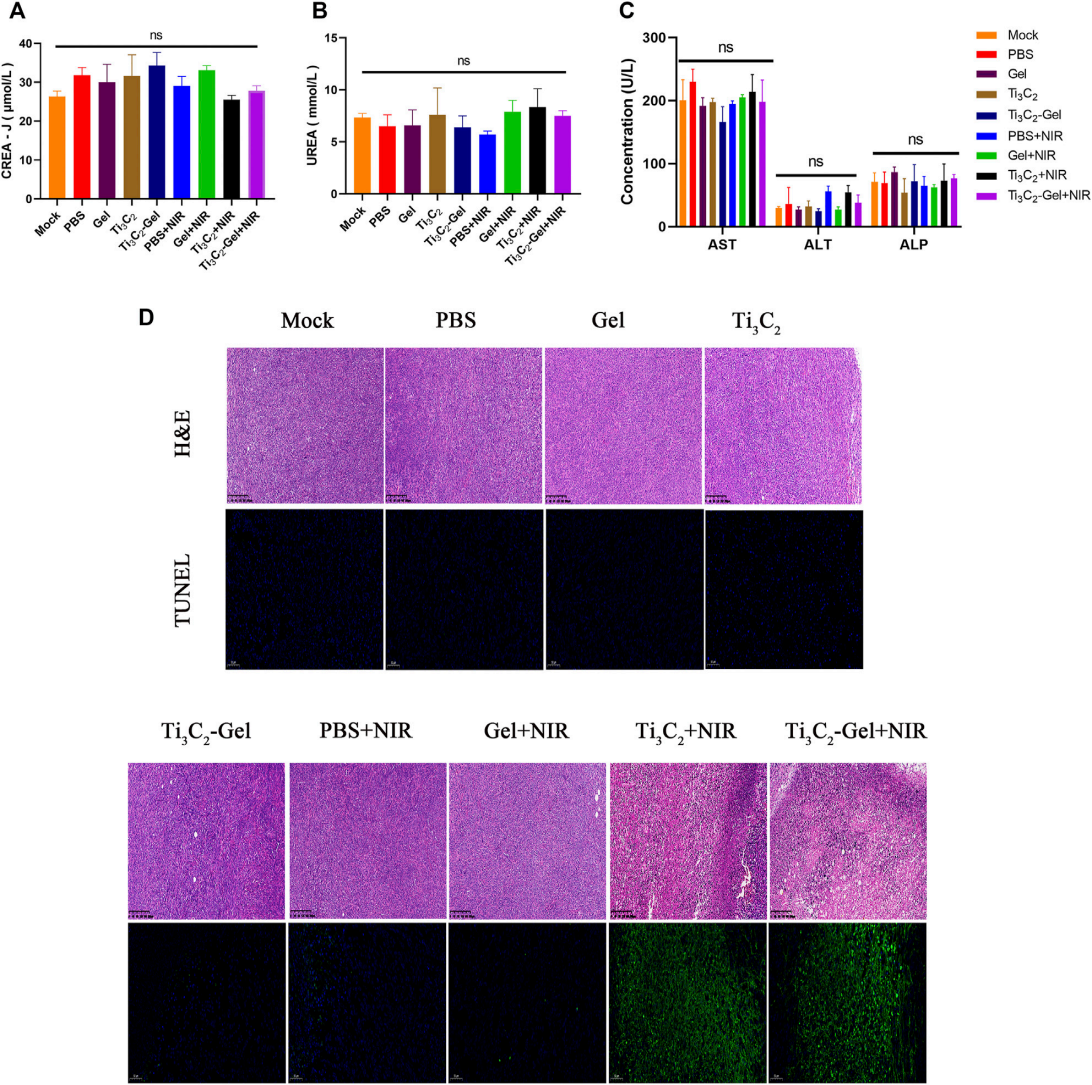
*In vivo* safety evaluation. **(A–C)** Blood biochemistry analysis (including indicators of liver and kidney function) of mice after various treatments. *n* = 3/group, mean ± SD. **(D)** Hematoxylin and eosin staining and TUNEL staining of tumor sections from different treatment groups.

These superior antitumor effects were verified by H&E and TUNEL staining of the tumor sections (Figure 6D). The tumor tissues in the Ti_3_C_2_-Gel + NIR group showed more obvious histological damage compared to the typical tumor structure in other groups, such as partial tumor destruction and necrotic response, indicating significant apoptosis. The body weights of the mice were measured to evaluate the systemic toxicity of the treatments. No obvious body weight loss was observed in these groups during treatment, indicating the biocompatibility of the Ti_3_C_2_ and F127 hydrogels (Figure 5G). In addition, the indicators of liver and kidney function in all mice were detected using a biochemical analyzer. The blood biochemical indicators of mice treated with Ti_3_C_2_-Gel showed no obvious differences from those of other control groups, suggesting healthy liver and kidney functions (Figures 6A–C). Similar to previously reported results (Chen et al., 2019a; Fu et al., 2019; Qin et al., 2019), the F127 hydrogel delayed the release of the loaded drug and avoided accumulated toxicity. The *in vivo* results reveal the potential of the Ti_3_C_2_-Gel system to exert photothermal therapeutic effects with high biocompatibility and biosafety.

## Conclusion

In summary, we constructed an injectable and biodegradable theranostic system based on Ti_3_C_2_ nanoparticles and Ti_3_C_2_-Gel for robust PTT. The Ti_3_C_2_ nanoparticle showed a shuttle structure with a diameter of approximately 50 nm, making it useful as a PTA for PTT. As a thermosensitive hydrogel approved by the FDA, F127 is combined with Ti_3_C_2_ nanoparticles to form a versatile photothermal gel system through a simple mixture. In addition, *in vivo* and *in vitro* experiments showed that the gel system has an excellent therapeutic effect against breast cancer. The Ti_3_C_2_-Gel can elevate the temperature of tumor tissues to ablate 4T1 cancer cells upon relatively mild laser exposure (40–50°C). The Ti_3_C_2_-Gel system not only has excellent photo-heat conversion and photothermal stability under repeated irradiation but also can form gels *in situ* in the body to increase the retention time and reduce the toxic side effects of the nanoparticles. As a result, this injectable Ti_3_C_2_-Gel system provides a translational paradigm for the photothermal treatment of breast cancer.

## Data Availability

The raw data supporting the conclusion of this article will be made available by the authors, without undue reservation.
